# A 3D digitization system for conventional radiation therapy simulation

**DOI:** 10.1120/jacmp.v6i3.2093

**Published:** 2005-08-17

**Authors:** Hsiao‐Ming Lu

**Affiliations:** ^1^ Department of Radiation Oncology Massachusetts General Hospital, Harvard Medical School 30 Fruit Street Boston Massachusetts 02115 U.S.A.

**Keywords:** radiation therapy, conventional simulation, 3D digitizer, body contour

## Abstract

While the majority of patients receiving external beam radiation therapy treatment are planned by CT simulation, a significant number of them are still planned using conventional simulators for various reasons. The information‐collection process in a conventional simulation is often fragmented and done with primitive tools. For example, in many institutions body contours are still acquired using solder wires and tracing paper, a time‐consuming and error‐prone procedure. We have developed a 3D digitization system to assist the information‐acquisition process at conventional simulations. The system consists of an infrared camera assembly, a wireless digitizer probe, and Windows‐based software. The system can provide 3D coordinates of any points in space accessible by the probe with submillimeter accuracy. It can be used to capture body contours, to record the coordinates of portal points, and to take various measurements for dose calculations as well as for patient setup. The software can display all the captured data together with the planned treatment fields, providing a comprehensive geometric verification of the treatment configuration. The system can also transfer all the information to dose‐planning programs in DICOM‐RT format, providing an integrated information flow from simulation to dose planning.

PACS: 87.53.Vb, 87.53.Xd

## I. INTRODUCTION

An important step in the simulation procedure for radiation therapy (RT) planning is to acquire treatment‐related geometric information. This includes measurements required for dose calculations, for example, patient body surface contours, tissue thickness, and tissue deficiencies. Treatment‐related information also includes collecting information for setting up treatment fields on patients, for example, the optical distance indicator (ODI) reading for the field and the translation of isocenter relative to certain tattooed points on the patient's skin surface. More and more RT patients are now planned by CT simulators, providing comprehensive and accurate 3D information for dose calculation and patient setup information.^(^ ^1^ ^)^

However, a substantial number of cases are still planned using conventional simulators, either because a CT simulator is not available at the facility or because the limited dimension of the CT aperture cannot always accommodate the particular body posture optimal for RT treatment. The information‐acquisition process at the conventional simulator is often fragmented and done with primitive tools. For example, a substantial number of clinics still use solder wires to obtain body contours. With this method, a wire is first pressed onto the patient's body, conforming to the skin surface; then the wire is traced on paper to produce the contour. A number of marks are made on the contour to indicate the projection points of the field center, field border, etc., so that the contour can be properly oriented. The paper contour is then digitized into the treatment‐planning computer for dose calculation. The process is slow and inaccurate. It is particularly troublesome when, after the contour is entered into the computer, one finds that it is not consistent with the simulated treatment fields. Because of the laborious nature of the method, multiple contours are seldom taken, even for cases where they may lead to more accurate dose calculations.^(^ ^2^ ^)^ More accurate contouring systems are available commercially, for example, the contour plotter (Med‐Tec Corporation, Orange City, IA).^(^ ^3^ ^)^ However, these devices either involve bulky equipment or do not provide a digital interface with treatment‐planning systems, and thus are not widely used.

In a previous communication, we reported the use of a 3D digitizer to assist the CT‐simulation procedure.^(^ ^4^ ^)^ The system can provide a virtual light field projection over the patient's body so that the portal points, for example, field center and field corners, of the treatment field determined by virtual simulation can be located on the patient's skin surface and properly tattooed for patient setup. In this work, we report on a system that aids the information acquisition during conventional simulation procedures. By interfacing the 3D digitizer with Windows‐based software, the system can take patient contours as well as the coordinates of skin surface marks from which patient setup information can be derived. With the voice guidance and a template system customized to each treatment site, the system is convenient and highly efficient. More importantly, the simulated treatment fields can be entered into the software so that their geometric consistency with the contours and points can be verified immediately. The system can transfer the fields, the contours, and the points to the treatment‐planning system via the DICOM format, providing a comprehensive and self‐consistent information package.

Our system was developed originally using a 3D sonic digitizer, the same as the device used in the virtual light field application for CT‐simulation.^(^ ^4^ ^)^ The accuracy of the sonic digitizer is affected by environmental factors, such as large variations in the air temperature across the digitization volume. For example, it can become unreliable when the device is near a strong cooling fan. Moreover, it is difficult to permanently position the large triangular detector frame in the simulator room without constant interference from the simulator gantry. As a result, at each use the frame has to be temporarily mounted onto the simulator gantry. We recently replaced the sonic digitizer by an optical digitization device with an infrared‐tracking camera system. Compared with the sonic digitizer, the optical system is advantageous in that it gives better and stable measurement accuracy and has a wireless digitizer probe. In addition, with a large active volume at a substantial distance from the camera assembly, the cameras can be mounted permanently without any interference from the simulator gantry. We have also adapted our virtual light field application^(^ ^4^ ^)^ to the optical digitization system. Both systems have been implemented for clinical use.

## II. METHODS

Since the present work shares many components with the virtual light field application reported earlier,^(^ ^4^ ^)^ these components will not be discussed here in detail. Instead, we focus on the development of the optical digitizer probe, the main software functions, and the measurements to evaluate the overall accuracy of the system for contouring.

### A. System configuration

**Figure 1:**
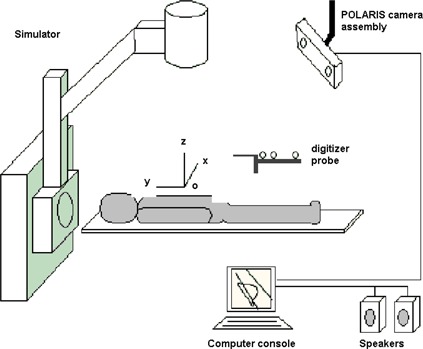
System components and configuration at a conventional simulator. The POLARIS infrared camera assembly is installed on the ceiling at a 45° angle from the isocenter. The coordinate system is defined such that if the patient is supine with the head toward the simulator gantry, the *x*‐axis points from the right to the left, the *y*‐axis from inferior to superior, and the *z*‐axis from posterior to anterior.

Figure 1 shows the system configuration and the various components at the conventional simulator. We use a commercial optical tracking system, POLARIS, with two infrared cameras mounted side by side on a frame.^(^ ^5^ ^)^ Surrounding each lens are arrays of diodes that emit infrared light during the operation. When a sphere (1 cm diameter) coated with infrared‐reflective material is introduced into the view of the cameras, the reflected light is captured by both cameras, and the coordinates for the center of the sphere can be calculated. The active tracking volume is shaped as a cylinder with a diameter of 1 m. Particularly helpful is the fact that the active volume is centered at 2.4 m from the camera frame. This allows one to position the camera just below the ceiling in front the simulator, 45° above the isocenter level. This arrangement centers the active tracking volume right at the isocenter of the simulator, where most of data acquisition takes place.

### B. Digitizer probe and the virtual trigger algorithm

The POLARIS system can track spherical infrared‐reflective markers with high accuracy (rms=0.35 mm).^(^ ^5^ ^)^ When these markers are mounted on a probe with a rigid body shape and a known geometry, the position of the probe tip in space can be calculated from the coordinates of the markers captured by POLARIS. Such probes have been used widely in image‐guided surgery and biopsy, where infrared markers are mounted on a surgical probe so that the position of the probe tip is continuously calculated and displayed relative to the patient's anatomy on the computer screen to guide the operation.^(^ ^5^ ^)^ However, these tracked probes are not suitable for digitizing data in the present application, because the coordinates of the tip are reported continuously to the PC workstation at all times, irrespective whether or not the probe tip is at the point to be digitized. The software program would not be able to determine which reported coordinates by the camera system are the intended data input.

A workaround may be possible by timing the digitization. For example, for digitizing a point, the software can specify a time window of a few seconds for the data entry, so that only the coordinates reported by the camera system within this time window would be kept as the position for the digitized point. Then if the operator places the probe tip at the point to be digitized before the time window opens and removes it only after the time window closes, the program would then be able to capture the correct data. However, such an approach may not be flexible enough for clinical use. While it may be workable for taking a few points, it would be impractical for taking long contours, particularly with unanticipated interruptions.

Ideally, the digitizer probe should be equipped with a trigger. Then the operator can signal the computer to accept the data reported from the camera as the valid coordinates for the digitized point of interest. This can easily be accomplished by using a simple electric trigger wired to the computer, but the probe would no longer be wireless, a very convenient feature for working around patients. Therefore, we developed a digitizer probe with a trigger that can signal the computer, not by hard wire, but by an algorithm in the program, thus a virtual trigger.

**Figure 2:**
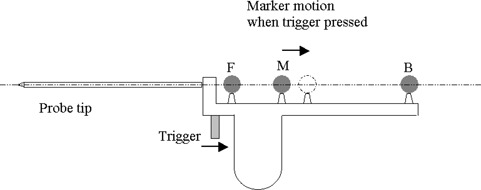
The basic design of the digitizer probe with the virtual trigger. All the spherical reflective markers (F, M, and B) are centered on a straight line that passes through the tip of the probe. When the trigger is pressed, the marker M moves back 5 mm.

Figure 2 shows our design of the digitizer probe. Three spherical markers are positioned along the axis of the probe tip. The front and back markers, F and B, are fixed to the probe frame, while the middle marker M is connected to the trigger. When the trigger is pressed, marker M can move back 5 mm along the axis. The markers are positioned such that even when the trigger is pressed, the middle marker is still closer to the front marker than to the back marker by more than 5 mm.

The algorithm works as follows. When the probe is in the active tracking volume of POLARIS, the camera system reports the coordinates of three detected markers to the workstation. Based on the facts that the three markers have to be on a straight line and the middle marker has to be closer to the front marker than to the back marker, the program can identify the front, back, and middle markers and assign their positions as $r_{F},r_{B}$, and $r_{M}$. Since the probe tip and the three markers are on a straight line, the tip position can be calculated by linear extension as

(1) $$r_{T}=r_{B}+(r_{F}-r_{B})d_{TB}/d_{FB},$$

where $d_{\text{TB}}$ and $d_{\text{FB}}$ are the known distances from back marker to the tip and from the back marker to the front marker, respectively.

During the tracking, the program continuously calculates the distance from the middle marker to the front marker $d_{\text{FM}}=|r_{F}‐r_{M}|$, and compares it to the value for the distance when the trigger is not pressed $\left(d_{\text{FM}}^{0}\right)$. When the difference $d_{\text{FM}}‐d_{\text{FM}}^{0}$ is greater than an appropriate threshold value ϕ(=3 mm), the program understands that the trigger has been pressed, and the current position of the probe tip as calculated by Eq. (1) should be kept as data input.

With the virtual trigger, the data can be captured conveniently. To digitize a point on a patient, one places the digitizer tip at the point and presses the trigger once. To enter a patient's body contour, one can just cruise the digitizer tip along the patient's skin surface while holding the trigger.

The coordinates obtained for the probe tip are given in the POLARIS coordinate system. These coordinates must be transformed to the isocenter coordinate system for the simulator to be consistent with the coordinate system in the treatment‐planning software. Reference 4 gives a detailed description for obtaining such a transformation. The same method applies in the present case and therefore will not be discussed further.

### C. Software functions

**Figure 3:**
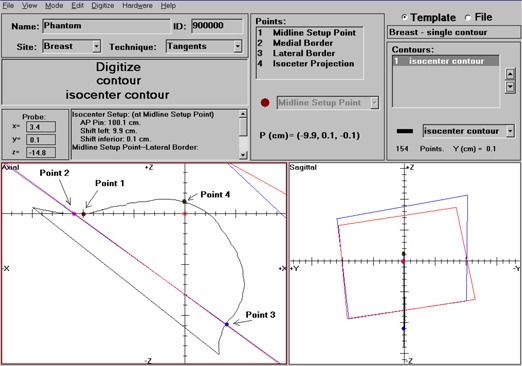
Software user interface for the simulation of a breast treatment using tangential fields. The the digitized contour and points both in an axial and a sagittal view, as well as the field border projections. It also shows the setup parameters derived from the digitized points to be compared with the actual measurements using, for example, ODI reading and rulers.

Figure 3 shows the software user interface when performing digitization for a left breast treatment using tangential fields. The software organizes the digitized data into two categories: points and contours. The points include the treatment field‐related points, for example, field center and field borders, as well as setup‐related points, for example, the places to take the ODI readings. The points can also be used to obtain simple geometric quantities, such as separations, for performing simple dose calculations. They can also be used to generate simple setup parameters that were traditionally measured with rulers and/or special measurement devices, that is, a breast bridge (Med‐Tec Corporation, Orange City, IA). The contours may be used for dose calculations in a treatment‐planning system or for simple calculations, for example, compensator evaluation.

The digitized contours and points are displayed together with the field border projections of the tangential fields in both axial and sagittal views in Fig. 3. For this particular type of treatment, four points on the isocenter plane were digitized: the medial and lateral border projections of the tangent fields, the isocenter projection on the anterior skin surface, and the midline setup point, which is the intersection between the patient's anterior midline and transverse plane at the isocenter. Using the coordinates of these points, the program calculated all the geometric parameters required for patient setup at the treatment unit. These included the ODI reading (AP Pin) to the tattooed midline setup point, the horizontal shifts to the isocenter position, as well as the so‐called chest wall angle used to ensure that the patient was not rotated.^(^ ^6^ ^)^

The two orthogonal views (axial and sagittal) allow one to capture contours either in the transverse planes or in the sagittal planes. A sagittal contour can be useful in some cases, for example, in determining the angle of a compensator in the longitudinal direction.

Treatment field parameters can be entered, and field projections are displayed together with the digitized points and contours (Fig. 3). These displays provide a consistency check between the treatment fields, the captured contours, and the points marking the projections of the fields on the patient skin surface.

All the information collected by the system (Fig. 3), that is, the points and body surface contours, treatment fields, patient's name and identification number, can be exported as files in the DICOM format.^(^ ^7^ ^)^ These files can then be imported into a treatment‐planning system, for example, Eclipse.^(^ ^8^ ^)^ The treatment fields are saved directly in a RT Plan file. The body surface contours and the points are, however, used to build a series of CT image files covering the region of interest with user‐selected spacing between the slices. The CT density in the areas included by the contours is set to the CT density of water, while the rest is set to that of the air. The location of the points is shown by setting the CT density for a 2×2 mm region centered at the point position to the CT density of lead. The inset in Fig. 6 displays such an image and field projections for a left breast treatment after the DICOM files have been imported into the planning system.

In addition to the basic functions of capturing points and contours, the software also has a number of features that are essential for routine clinical use, similar to the virtual light field application developed earlier.^(^ ^4^ ^)^ These include the template system, the digitization sequence, the remote control, and the voice guidance. The template system is based on the fact that many treatment sites use standard treatment techniques; thus, the points and contours to be digitized are well defined. For these sites, templates can save time and expedite the procedure significantly. Each template contains a default list of points and contours to be digitized, as well as a default set of treatment fields associated with the specific treatment technique for the site. It is also embedded with specific methods that use the digitized points and field information to derive treatment and/or setup parameters only relevant to this treatment site, for example, anterior ODI reading and lateral separation. The template also organizes the points and contours into a digitization sequence. In the sequence mode, the program automatically receives the digitization input and moves from point to point and from contour to contour. The remote control allows the operator to make a menu selection by using only the digitizer probe, so the operator can execute the program without having to constantly walk back and forth to the computer console to use the mouse or the keyboard. The system provides voice instructions through the speakers to guide the operator. At each step of the digitization sequence, the program announces the item to be digitized and the specific instructions for the digitization, so that the correct points or contours are digitized.

### D. System accuracy evaluation

The POLARIS camera system is highly accurate in capturing the coordinates of spherical reflective markers mounted on the digitizer probe throughout the entire tracking volume (rms=0.35 mm).^(^ ^5^ ^)^ However, the overall accuracy of our system also depends on the geometric accuracy of the digitizer probe, the accuracy of the calibration procedure to build the coordinate transformation, etc. Moreover, because the digitizer probe tip has to be dulled to avoid injuring the patient during digitization, it is not always easy to place the probe tip precisely at the point to be digitized. The accuracy in placing the probe tip may also depend on the orientations of the probe, as well as the visualization of the point on the patient.

**Figure 4:**
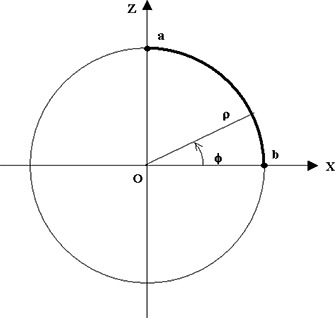
Coordinate system for the accuracy test using a cylindrical phantom. The heavy line indicates the quadrant of the cycle digitized as the contour.

In order to obtain a basic evaluation of the overall accuracy of the system for contouring, we performed measurements at the simulator using a cylindrical plastic phantom with known dimensions. The cylinder is positioned at the isocenter of the simulator with its main axis along the longitudinal direction, that is, the *y*‐axis of the room coordinate system (Fig. 1). Then the intersection of the cylindrical surface and the vertical side lasers forms a circle with a known radius (16 cm) centered at the isocenter and in the *xz*‐plane. Following the laser projections, we digitized a quadrant of this circle as a body contour (Fig. 4) with a reasonable tracing speed (~2 cm/s). We also digitized two points (a and b) separately to evaluate the accuracy of coordinate capture for individual points. The measurement was repeated 10 times for the same setup.

Deviations from zero were calculated for the *y*‐coordinates of the contour points. These gave the error of measurements in the direction perpendicular to the contour plane. For comparison within the contour plane, we converted the *x* and *z* coordinates of the points to a cylindrical coordinate system defined by φ=arctan(z/x) and $\rho ={\left(x^{2}+z^{2}\right)}^{1/2}$, as illustrated in Fig. 4. Since the contour is part of a circle with radius 16 cm, the deviation in the radial direction at each point is Δρ=ρ‐16 cm. At each angle, we can calculate these deviations for all 10 measurements by linear interpretation. Deviations were also calculated for the two points a and b against their known coordinates (0, 0, 16) and (16, 0, 0), respectively.

## III. RESULTS OF SYSTEM ACCURACY EVALUATION

**Figure 5:**
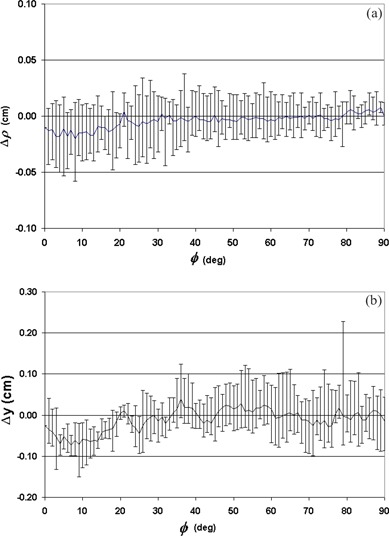
Deviations of digitized contour points from their expected values in (a) the radial direction and (b) the *y*‐direction. The lines show the average values over the 10 measurements, while the error bars represent the range of the deviations.

(Figure 5a) shows the average (line) and the range (error bars) of the deviations of the contour points in the radial direction (Δρ) as a function of angle (φ). All the measurements are basically within ±0.05 cm. The deviations in the direction perpendicular to the contour plane (*y*‐direction) are given in (Fig. 5b), showing a larger range (±0.10 cm) of variations. For points a and b, all measurements are within ±0.06 cm, except for the *y*‐coordinates of point a, where the maximum error was 0.08 cm, and the standard deviation was 0.05 cm.

**Figure 6:**
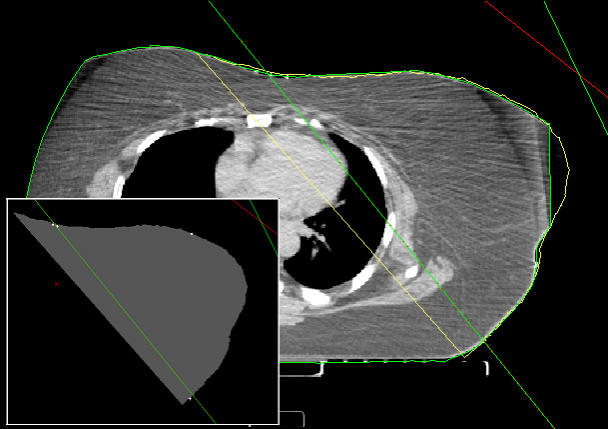
A case example of a left‐breast treatment where CT‐generated body contours (green) did not include the entire breast. The dose calculation had to be carried out using the body contours obtained from the 3D digitization system (yellow).

## IV. DISCUSSION

While the majority of patients are now planned by CT simulation, a significant number of them are still planned using conventional simulators. For these patients, the system presented here is an accurate and integrated tool for acquiring and transferring treatment‐related geometric information to the treatment‐planning system. Its efficiency in taking multiple body surface contours allows the routine use of multiple contours for treatment sites where multislice dose planning was shown to be beneficial.^(^ ^2^ ^)^ The system can also be useful for the rare occasions when the CT images do not include all the patient body surfaces due to patient obesity or obstructions of the immobilization devices. In these cases, the images can still be used for optimizing the geometric placement of the treatment fields, but they cannot provide complete body surface contours for dosimetric calculation and optimizations. Figure 6 shows such an example where an obese patient was simulated for left‐breast treatment. While the CT images provided definitions of lung and cardiac tissues to allow optimal placement of the tangential fields, part of the left breast was not imaged properly to be included in the auto‐segmented body surface contour (green). Instead, the dose calculation and wedge optimization had to be performed using the contours taken by the 3D digitization system (yellow). The inset in Fig. 6 shows the view of the isocenter slice after the points and contours have been imported into the planning system as discussed earlier.

The accuracy test presented here was intended to measure the intrinsic accuracy of the 3D digitization system. For that reason we used a solid phantom with exact known dimensions. We also compared the digitized points and contours with those generated from CT scans of phantoms. However, such comparisons included the errors generated during the CT‐imaging process, for example, phantom repositioning, image reconstruction, and segmentation. Comparisons were also made for real patients, as the example shown in Fig. 6. The results are generally satisfactory, considering the additional uncertainties from patient repositioning and body surface motions due to respiration.

When tracing the contour along the laser line on the slippery cylindrical phantom surface during the accuracy test, it was difficult to keep the probe tip from moving in and out of the laser line. This contributed to the errors in the contour points in the direction perpendicular to the contour plane (*y*). In the radial direction (*ρ*), however, because the probe tip is always in contact with the surface, the error came from only the intrinsic uncertainty in the system and was therefore smaller.

The errors from all the measurements were well within the tolerances for routine dose planning and treatment setup parameter calculations. However, this represented only the best scenario, since the test was on a solid phantom with a hard surface where we could basically slide the probe tip. On the soft skin surface of a real patient, the probe tip may depress into the skin surface and generate larger contour errors. Clearly, the accuracy will then depend on the operator's skills, which can be improved through training and practice.

The system has been in use in our clinic since 1999, starting with the sonic digitizer and then the infrared tracking setup after 2001. The most appreciated aspect of the system from the point of view of the simulation therapists is that all the geometric information; for example, contours, points, and setup parameters, are automatically integrated and verified for consistency as soon as they are taken during the simulation procedure, so that any mistake or mismatch can be corrected before the patient leaves.

Although we used a POLARIS infrared‐tracking camera in our setup, the software and the algorithms presented here can be adapted to any spatial tracking device. Particularly useful would be to adapt the system to those already in use for other radiotherapy functions, for example, the real‐time position management (RPM) respiratory gating monitor system.^(^ ^9^ ^)^ It has been shown that single‐camera systems such as RPM can track the orientation and position of rigid bodies, for example a digitizer probe, by using multiple non‐coplanar reflective markers.^(^ ^10^ ^)^

## V. CONCLUSION

We have developed a 3D digitization system for the acquisition of treatment‐related geometric information at conventional simulations for radiation therapy planning. Based on infrared‐tracking technology, the system can capture 3D coordinates of any points in space accessible by a wireless probe with submillimeter accuracy. With software features such as templates, digitization sequencing, remote control, voice guidance, and DICOM format output, the system is an efficient and accurate tool to obtain body contours, portal points, and any other geometric measurements for treatment planning and treatment setup. The overall accuracy of the system has been evaluated with acceptable results.

## ACKNOWLEDGMENTS

The author is grateful to Dr. Lee Chin for general guidance, support, and many helpful discussions. The author wishes to thank Frank Cardoza and Lucy Li, M.S., for helping with the hardware installation and engineering. The author also wishes to thank the department of radiation oncology at Brigham and Women's Hospital, Boston, for supporting the project.
